# Focused ultrasound to displace renal calculi: threshold for tissue injury

**DOI:** 10.1186/2050-5736-2-5

**Published:** 2014-03-31

**Authors:** Yak-Nam Wang, Julianna C Simon, Bryan W Cunitz, Frank L Starr, Marla Paun, Denny H Liggitt, Andrew P Evan, James A McAteer, Ziyue Liu, Barbrina Dunmire, Michael R Bailey

**Affiliations:** 1 Center for Industrial and Medical Ultrasound, Applied Physics Laboratory, University of Washington, 1013 NE 40th Street, Seattle, WA 98105, USA; 2 Department of Comparative Medicine, University of Washington School of Medicine, 1959 NE Pacific Street, P.O. Box 357115, Seattle, WA 98195, USA; 3 Department of Anatomy and Cell Biology, Indiana University School of Medicine, 635 Barnhill Dr., MS 5055, Indianapolis, IN 46202, USA

**Keywords:** Injury threshold, Kidney stones, Ultrasonic propulsion

## Abstract

**Background:**

The global prevalence and incidence of renal calculi is reported to be increasing. Of the patients that undergo surgical intervention, nearly half experience symptomatic complications associated with stone fragments that are not passed and require follow-up surgical intervention. In a clinical simulation using a clinical prototype, ultrasonic propulsion was proven effective at repositioning kidney stones in pigs. The use of ultrasound to reposition smaller stones or stone fragments to a location that facilitates spontaneous clearance could therefore improve stone-free rates. The goal of this study was to determine an injury threshold under which stones could be safely repositioned.

**Methods:**

Kidneys of 28 domestic swine were treated with exposures that ranged in duty cycle from 0%–100% and spatial peak pulse average intensities up to 30 kW/cm^2^ for a total duration of 10 min. The kidneys were processed for morphological analysis and evaluated for injury by experts blinded to the exposure conditions.

**Results:**

At a duty cycle of 3.3%, a spatial peak intensity threshold of 16,620 W/cm^2^ was needed before a statistically significant portion of the samples showed injury. This is nearly seven times the 2,400-W/cm^2^ maximum output of the clinical prototype used to move the stones effectively in pigs.

**Conclusions:**

The data obtained from this study show that exposure of kidneys to ultrasonic propulsion for displacing renal calculi is well below the threshold for tissue injury.

## Background

The clinical uses of ultrasound (US) span both diagnostic and therapeutic applications. This broad range of applications is due to the variety of bioeffects that can be elicited in tissue with US. The potential for tissue damage resulting from US has resulted in a need for safety guidelines to be established. Although guidance on the safety of diagnostic US was initiated in the 1970s, early discussions focused only on thermal bioeffects. It was not until the late 1980s that the safety of non-thermal mechanics was considered [1,2]. Despite the decades of research on the bioeffects of US, safety guidelines for therapeutic US have yet to be established [3], and treatment levels that lie between traditional diagnostic and therapeutic ultrasound categories have not been fully addressed. With the emergence of new applications utilizing a wide range of US systems, including diagnostic/therapeutic hybrids such as the Verasonics system (Redmond, WA, USA) [4], patient safety needs to be carefully evaluated for these in-between exposures. One such new application involves using US to expel renal calculi [5-7].

The global prevalence and incidence of renal calculi is reported to be increasing [8], with the recent National Health and Nutrition Examination Survey (NHANES) reporting a prevalence of 1 in 11 in the USA [9]. Shockwave lithotripsy (SWL) remains the principal treatment of symptomatic renal calculi (National Kidney Foundation) despite the tissue damage that can occur as a result [10-12]. Stone fragments are often left after SWL, which can act as nuclei for the formation of new stones, resulting in the need for further intervention or retreatment.

As such, ultrasonic propulsion was recently invented to use ultrasound to reposition kidney stones [5-7,13,14]. Application includes expelling not only residual fragments from the kidney but also *de novo* stones, accessing stones during surgery, and dislodging large emergent obstructing stones [14]. Pulses with maximum intensity of 2,400 W/cm2 have been used to reposition stones in animals effectively [5] and without observed injury [5,13].

The goal of this study was to evaluate acoustic intensities below which the ultrasonic propulsion system may be safely operated to reposition kidney stones. A custom research device was used to treat surgically exposed kidneys over a wide range of intensities. A threshold for injury was established by applying the plateau statistical model to the tissue evaluation. The results were compared to conventional lithotripsy output intensities and the output intensities used in a clinical simulation of treatment on a porcine model [5]. The results are not an exhaustive parameterization of safe outputs but an investigation of safety issues relevant to the outputs used in stone relocation, which may have relevance to other ultrasound applications as well as future clinical development.

## Methods

### Ultrasound device

These studies used a custom-built experimental ultrasound system [6,15]. In brief, the device consists of a 6-cm diameter, 2-MHz, eight-element annular array curved to fit a natural focus of 6 cm (H-106, Sonic Concepts, Bothell, WA, USA). An SC-200 radiofrequency synthesizer (Sonic Concepts, Bothell) provides eight channels of phase-delayed signals that are amplified by individual custom-modified 100-W IC-706MIKIIG amplifiers (Icom®, Bellevue, WA, USA) to excite the eight elements of the array. The focal depth of the treatment could be adjusted from 3.5 to 9.5 cm by using software written in MATLAB® (Mathworks, Waltham, MA, USA) to control the relative phase delay of each element. The focus was maintained between 1 and 1.5 cm below the kidney surface, which corresponds to a 6 or 6.5 cm total depth. Treatments were guided with a coaxially aligned P4-2 imaging transducer and an HDI-5000 Ultrasound system (Philips Ultrasound, Bothell, WA, USA). The transducer surface was kept cool by circulating water set to 8°C through the coupling cone using a modified water chilling system (EW-12108-10, Cole-Parmer®, Vernon Hills, IL, USA). The treatments were guided with a coaxially aligned P4-2 imaging transducer and an HDI-5000 ultrasound system (Philips Ultrasound).

### Treatment exposures

**Figure 1:**
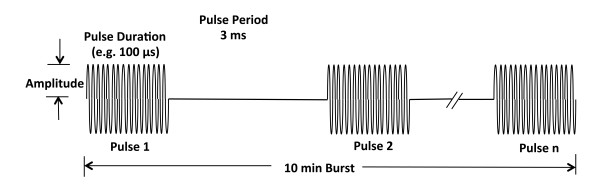
**Schematic of the treatment bursts.** The 3.3% duty cycle consists of a 100 μs long burst of pulses repeated every 3 ms over the 10-min treatment duration.

The research device was used to deliver a wide range of ultrasound doses (Table 1). Three different treatment protocols were implemented; all protocols had a total treatment time of 10 min. B-mode ultrasound imaging occurred throughout all exposures, but only the therapy exposures are discussed. The first protocol tested a 3.3% duty cycle burst consisting of a 100-μs long pulse repeated every 3 ms (Figure 1). This exposure protocol was identical to that used for the clinical simulation study [5]. The second protocol tested a 100% duty cycle (constant burst) output for 10 min (no time off). This protocol was intended to mimic a maximum dose treatment, in which the device was used in continuous operation. For the third protocol, one intensity was chosen and the duty cycle was varied by adjusting the length of the US burst while maintaining a 3-ms pulse period. The intensity was 10,700 W/cm^2^ in water, derated to 9,320 W/cm^2^ at a depth of 1 cm into the kidney tissue. This treatment mode evaluated the injury sensitivity to duty cycle at the maximum un-derated pressure of the clinical prototype, that is, assuming the ultrasound was focused into the kidney without attenuation from overlying tissues.

**Table 1:** Exposures used for renal parenchyma treatment

**Threshold intensity at normal duty cycle**		**Threshold intensity if on continuously**		**Sensitivity to duty cycle at maximum prototype intensity**	
**3.3% Duty cycle**		**100%Duty cycle**		***I***_**SPPA.3**_** = 9,320 W/cm**^**2**^	
***I***_**SPPA.3**_**(W/cm**^**2**^**)**	**Samples**	***I***_**SPPA.3**_**(W/cm**^**2**^**)**	**Samples**	**Duty cycle(%)**	**Samples**
0	29	0	13	0	13
930	12	470	3	2	7
2,530	14	930	6	6	5
4,090	10	2,530	7	10	8
6,030	10	4,090	8	25	3
9,320	12	6,030	5	50	5
12,230	11	9,320	4	100	4
16,620	14				
21,000	11				
26,130	10				

Exposures were based on the expected clinical exposure parameters (3.3% duty cycle), maximum duty cycle (100%) of clinical device, and for a range of duty cycles at an intensity with similar peak positive and negative pressures as the clinical device. The samples represent the number of tissue samples evaluated for injury at each condition.

**Figure 2:**
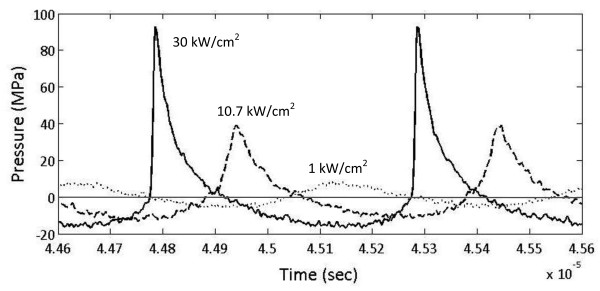
**Typical waveforms produced by the research device.** Measurements were performed in water showing the maximum achievable (solid line), minimum (dotted line), and a waveform approximating the peak positive and negative pressures generated by the clinical prototype (dashed line).

The intensity values in Table 1 and in this paper represent the spatial peak pulse average. The intensities were derated based upon the methods developed for non-linear high-intensity focused ultrasound (HIFU) waves [16-18] using a derating factor of 0.3 dB/cm/MHz, which is recognized by the FDA [19,20]. The maximum spatial peak pulse averaged intensity (*I*_SPPA_) that could be achieved with the research device was found to be 30,000 W/cm^2^ in water with a corresponding peak positive pressure of 96 MPa and a peak negative pressure of 16 MPa (Figure 2). This corresponds to a derated *I*_SPPA.3_ of approximately 26,000 W/cm^2^ at 1-cm tissue depth. The pressure waveforms showing the range of treatment intensities measured in water are provided in Figure 2 for comparison.

### Animal treatment protocol

The kidneys of the domestic swine were treated *in vivo* following a protocol approved by the Institutional Animal Care and Use Committee at the University of Washington. A total of 28 female pigs weighing 101–141 lbs were sedated with an intramuscular injection of telazol (4 mg/kg). Anesthesia was maintained using isofluorane (1.5%–2.5%) via endotracheal tube. The abdomen was opened, and the intestines were repositioned to one side to reveal the kidney that was to be treated. The overlying renal fascia was removed, and the abdominal cavity was filled with degassed phosphate buffered saline (PBS) for coupling of the transducer. The kidney was immobilized with wet gauze; care was taken to avoid placing the gauze in the US path.

The US transducer was positioned, and the renal parenchyma was targeted for treatment. The transducer has a water-filled cone that is 5 cm in length (as measured from the center of the curved transducer). The cone was placed directly on the kidney, and the transducer was programmed to a focal depth of 6 or 6.5 cm. The focal depth is programmable and controlled by the timing of the different elements of the eight-element annular array. This makes the focus 1–1.5 cm inside the kidney. The settings (Table 1) used for each exposure were randomly selected at each treatment spot. Up to seven distinct locations were treated in each kidney. The areas were kept treatment free for control samples. With the exception of the 100% duty cycle exposures, the US image was monitored during the treatment for appearance of echogenicity in the focal region. After each exposure, the kidney was inspected, and the treatment location was marked with histology ink. Any visible gross changes to the kidney surface were also noted.

In order to maximize the *in situ* intensity exposure and to accurately mark and analyze the treated tissue, the kidneys were immobilized and exposed directly to the US energy, rather than transcutaneously, as would be the standard protocol in humans. The two approaches are equated by the focal derated acoustic intensity. As noted in [13], output levels were insufficient to generate observable kidney injury with exposure through the skin and the corresponding acoustic attenuation. All the animals were euthanized upon completion of the experimental treatment.

### Injury evaluation

The kidneys treated at a duty cycle of 3.3% were perfusion fixed *in situ* before being removed for routine histological evaluation [21]. Individual treatment locations as indicated by the histology ink and control tissues were embedded separately in paraffin, and sections were stained with hematoxylin and eosin (H&E) and periodic acid-Schiff (PAS). This protocol is an established tissue preparation technique used to analyze the hemorrhagic lesion induced by SWL in pigs and also associated with mechanical effects of low duty cycle pulses [21]. Stained slides from the treated and control samples were randomized and reviewed by three independent experienced experts blind to the experimental conditions. Each reviewer provided histopathological descriptions of each slide. From these descriptions, the slides were scored according to a grading rubric developed by a veterinary pathologist (Table 2). The specimens that were given a score of 1 or above were considered to be injured. The results were therefore binary in nature for statistical analysis.

**Table 2:** Grading criteria for histological evaluation of the kidneys

**Score**	**Description**
0	No treatment associated lesions
1	Focal degenerative change including epithelial cell swelling, tissue hyperemia (congestion)
2	Degenerative change accompanied by focal regions of individual epithelial cell necrosis
3	Focal coagulative or liquefactive necrosis (emulsification) with hemorrhage

Samples with scores of 1 and above were considered to be injured.

The kidneys treated at a duty cycle of 100% and with a constant intensity of 9,320 W/cm^2^ were removed and immediately processed for preparation of frozen sections. The frozen sections were stained for nicotinamide dinucleotide diaphorase (NADH-d) to evaluate thermal injury [22]. Stained slides from the treated and control samples were randomized and reviewed by one experienced expert blind to the experimental conditions. Only one individual reviewed the NADH-d-stained slides as the reading was binary; areas with non-stained tissue indicated thermal damage and was marked as being positive for injury. This is an established preparation technique for analysis of porcine renal and hepatic injury from HIFU, which is associated with thermal effects for high duty-cycles or long duration pulses [22,23]. Since these studies used longer pulses more like HIFU than SWL, NADH staining was chosen.

### Statistics

For the 3.3% duty cycle data, inter-observer variability was evaluated using an intra-class correlation (ICC) with a 95% confidence interval before averaging across observers. The threshold for injury for all three sets of data (3.3% duty cycle, 100% duty cycle, constant intensity) was calculated using the plateau model. The threshold for the echogenicity of the 3.3% duty cycle group was also determined using a generalized plateau model since the outcomes were binary. The plateau model is a special case of the linear change point model, where the second slope is zero, which was tested and confirmed in analysis [24]. In the plateau model, the dependent variable, denoted as *y*, is related to the independent variable, denoted as *x*, in two different ways. The change point *x*0 defines when the relationship changes, which is referred as the threshold in this paper. For *x* > *x*0, *y* is linearly related to *x*. For *x* < *x*0, y is not affected by *x*. Instead, it stays flat (hence the term plateau). In this paper, *y* is the tissue injury and *x* is the intensity. For intensity below the threshold, there is basically no tissue injury; when the intensity is above the threshold, the injury increases with the intensity. Random intercepts were used to account for within-subject correlations. The threshold was selected by searching over candidate points, and model selection was performed using Akaike information criteria. Two-sided *p* < 0.05 were considered statistically significant. All analyses were performed using SAS 9.3 (SAS Institute, Cary, NC, USA).

## Results

### 3.3% Duty cycle

**Figure 3:**
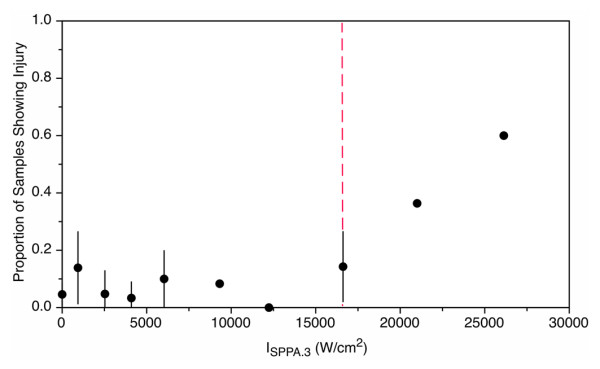
**Injury at 3.3%duty cycle.** Proportion of samples that show injury versus the spatial peak pulse averaged intensity. All exposures were at 3.3%. Dashed line indicates threshold. Error bars represent one standard deviation. When no error bars are observed, all evaluations were in agreement.

**Figure 4:**
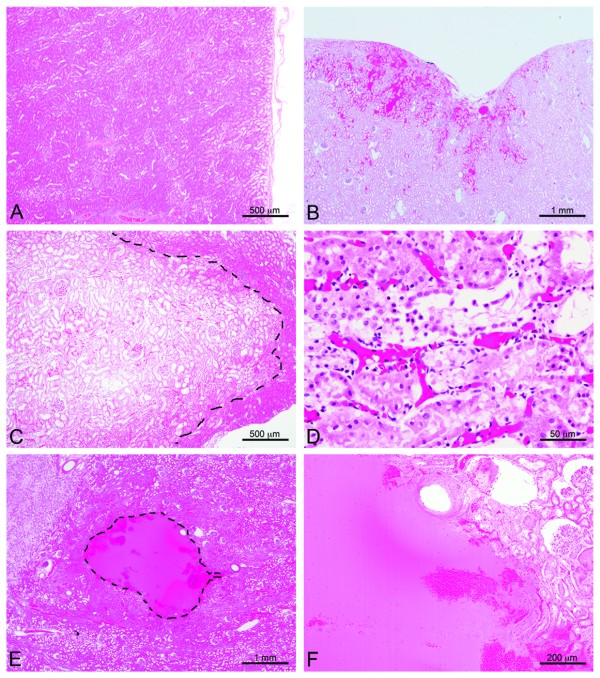
**Histological examples of injury.** Most histologic changes in this study were subtle consisting of mild cell swelling or tissue congestion and varied only slightly from the control tissue **(A)**. Modestly more significant lesions infrequently occurred below the threshold intensity and consisted of some focal congestion and hemorrhage along with individual cell necrosis and tissue compression evident here **(B)** in a single subcapsular site. Above the threshold, tissue injury was more pronounced. Large, focal pale region (dashed line, **C)** composed of degenerative epithelial cells surrounded by areas of tubular epithelial cell necrosis with sloughing of tubular lining cells **(D)**. At the extreme, there were distinct foci (dashed line) of liquefactive necrosis or emulsification **(E)** that on higher magnification **(F)** abruptly interfaced with more normal tissue and resulted in cavities which were filled with lysed and intact erythrocytes (hemorrhage).

The ICC between the reviewer scores was found to be 0.86 (95% 0.66–0.95), which means that the three reviewers were in near-perfect agreement. Consequently, the averages of the three reviewer scores were used for all subsequent analyses. Figure 3 shows a plot of the proportion of samples that showed histological injury versus the derated spatial peak pulse averaged intensity. The plateau model revealed a change point (threshold) at a derated intensity of 16,620 W/cm^2^, below which the probability of injury was less than 0.2. Below the threshold, histologic changes detected following this treatment protocol were relatively minor, consisting of background lesions, or focal tubular epithelial cell changes such as cell swelling consistent with a mild degenerative change. The vast majority of the samples were similar in appearance to the control samples (Figure 4A). Of the 69 tissue samples treated below the threshold (not including the controls), only 2 samples displayed an evidence of focal individual cell necrosis and/or hemorrhage. These lesions were typically superficial in nature (Figure 4B) and were not found at the targeted focus position in the parenchyma. Neither lesion showed evidence of emulsification (liquefactive necrosis). All other histological changes detected below the threshold were relatively mild, typically degenerative, and rarely involved individual cell necrosis. Above the threshold intensity, the lesions contained focal areas of emulsification, individual cell, and coagulative necrosis, which were frequently accompanied by hemorrhage (Figure 4C,D,E,F). Many of the lesions seen in the tissue treated above the change point were on the order of millimeters and could sometimes be seen in gross observation of the surface.

**Figure 5:**
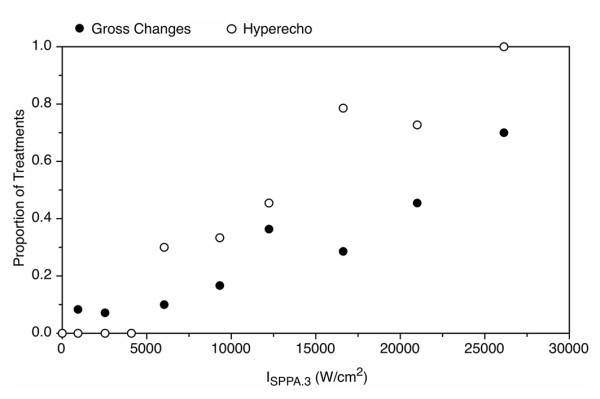
**Changes during the 3.3% duty cycle protocol.** Proportion of samples showing hyperechogenicity or gross changes versus the derated spatial peak pulse averaged intensity. All exposures were at 3.3%.

**Figure 6:**
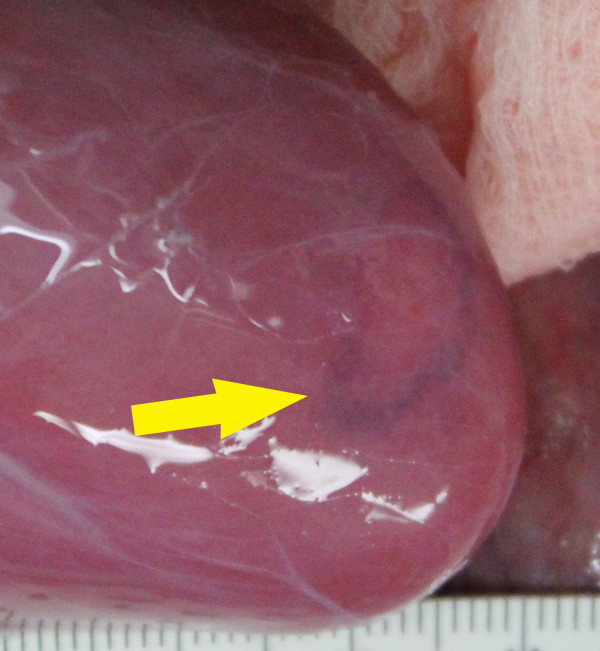
**Gross surface change.** Photo of a typical gross surface change after treatment (3.3% duty cycle; 26,130 W/cm^2^). Arrow indicates edge of surface reddening. The treatment direction was into the page. Scale bar in millimeters.

Although the proportion of gross changes observed immediately after treatment generally tracked with the pattern for the histological observations (Figure 5), gross surface changes did not necessarily correlate with histological injury, particularly below the threshold. Below the 16,620-W/cm^2^ threshold, the proportion of samples that showed gross changes was slightly higher than the proportion of samples with histological signs of injury. The majority of the gross changes observed included reddening or congestion (Figure 6). In many cases, the gross changes were not apparent after perfusion and on tissue sections.

**Figure 7:**
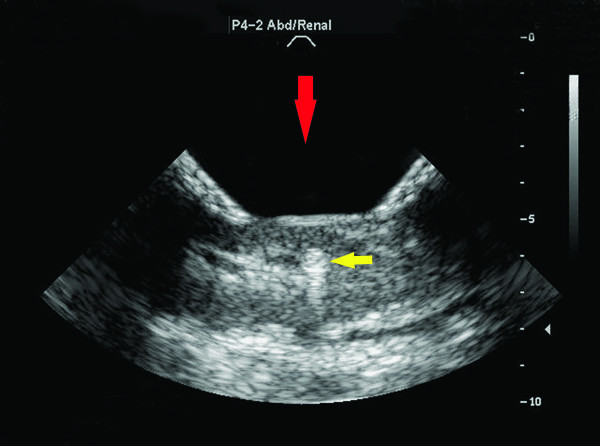
**Hyperechoic region during treatment.** Screen shot of a hyperechoic region (yellow arrow) observed in the kidney tissue during treatment (3.3% duty cycle; 26,130 W/cm^2^). Treatment direction was from the top (Red arrow). Scale in centimeters.

Hyperechogenic focal regions (Figure 7) were sometimes observed during treatment and usually appeared immediately after the start of the exposure. The proportion of treatments that exhibited focal hyperechogenicity generally tracked with the occurrence of histological injury (Figure 5). No hyperechogenic regions were observed at or below an intensity of 4,090 W/cm^2^. Above the histological injury threshold, the probability of observing a hyperechogenic region is greater than 0.5. Both the curves for gross changes and for hyperechogencity show (Figure 5) a rise in the proportion, showing hyperechogenicity or injury, respectively, to a level 0.5 or higher above 16,000 W/cm^2^, which is consistent with the threshold in Figure 3.

### 100% Duty cycle

**Figure 8:**
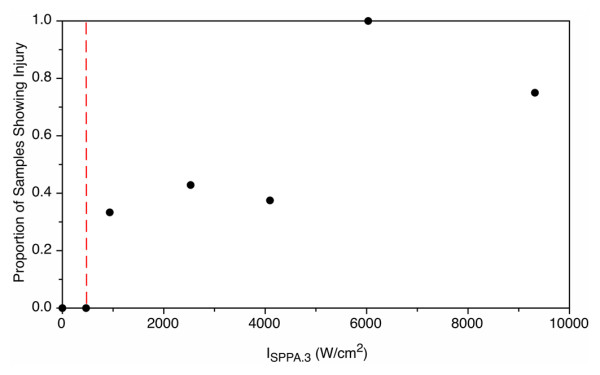
**Injury at fixed duty cycle.** Proportion of samples showing injury with increasing derated spatial peak pulse averaged intensity at a fixed duty cycle of 100%. Dashed red line represents the threshold.

**Figure 9:**
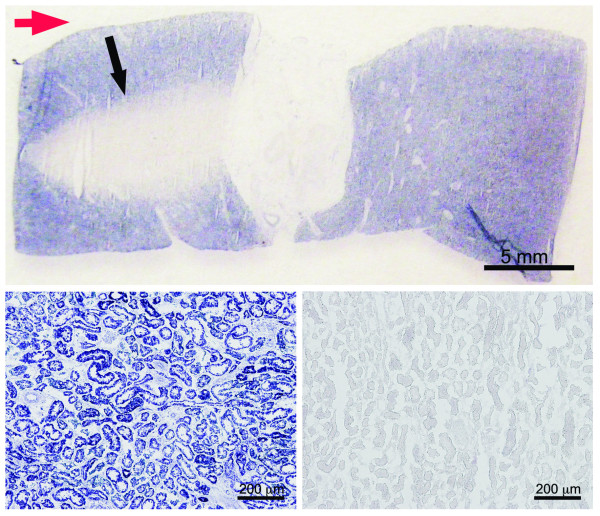
**NADH-d evaluation.** Example of full thickness (entire kidney) tissue section stained with NADH-d (top). Treatment location was from the left (Red arrow). Non-treated parenchyma stains purple/blue (bottom left); lesion is identified by non-staining (black arrow and bottom right). This tissue was treated at a duty cycle of 50% at a fixed derated spatial peak pulse averaged intensity of 9,320 W/cm^2^.

For the 100% duty cycle exposures, the plateau model determined a change point at a derated spatial peak intensity of 470 W/cm^2^ (Figure 8). Aside from the control samples, no other intensities were evaluated below this threshold with the NADH-d stain. Above this threshold, the lesions observed were on the order of millimeters to centimeters in size. Treated regions showed no evidence of staining (Figure 9). A large rise in the proportion of injury from 0.4 to 1 was observed at 6,000 W/cm^2^. At higher intensities, the lesions often extended the whole thickness of the kidney, and thermal lesions were visible on both the anterior and posterior surfaces of the kidney after treatment.

### Fixed elevated intensity

**Figure 10:**
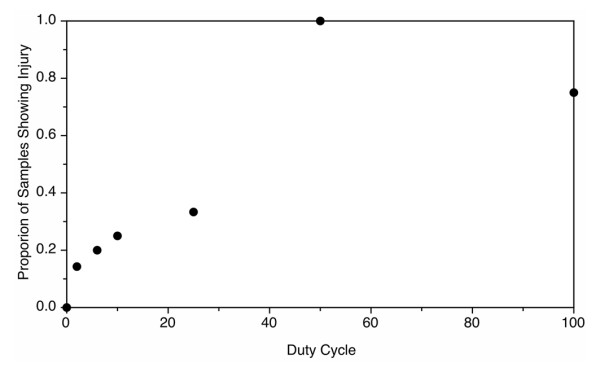
**Injury at a fixed intensity.** Proportion of samples showing injury with increasing duty cycle at a fixed derated spatial peak pulse averaged intensity of 9,320 W/cm^2^.

At a fixed intensity of 9,320 W/cm^2^, the plateau model determined a change point at a duty cycle of 2% (Figure 10). At this level, the probability of injury is below 0.2. There is another prominent rise in the curve at 50% duty cycle, where the probability first exceeds 0.5 and rises to 1. The probability of injury is rather low and insensitive to duty cycle up to 25%. As in the 100% duty cycle section, injury was identified with the NADH-d stain, which is shown in Figure 9.

## Discussion

In this study, the exposure range to move kidney stones by ultrasonic propulsion was expanded to explore thresholds for tissue injury. A duty cycle of 3.3% was selected specifically to compare with a clinical simulation previously performed in pigs [5]. At a 3.3% duty cycle, over a total of 10 min, the threshold for injury was found at 16,620 W/cm^2^. In the clinical simulation, stones were effectively moved using a 3.3% duty cycle, but with only 26 bursts of pulses that extended only for 1 s each and derated spatial peak intensities near 2,400 W/cm^2^ (through 7 cm of tissue). This is the derated intensity delivered transcutaneously to a depth of 7 cm. Thus, even this threshold is conservative, and the calculated injury threshold indicates that the intensity could be safely increased if more force was needed to, for example, detach a large stone from the tissue or to fragment a stone without the fear of generating tissue injury. Table 3 shows a comparison across acoustic exposures for urolithiasis and the injury thresholds.

**Table 3:** Parameter comparison for the clinical prototype for displacing renal calculi, shockwave lithotripsy, and diagnostic ultrasound

**Device**	***F***_**c**_**(MHz)**	**Pulse duration(μs)**	**Duty cycle**	***P***^**+**^**(MPa)**	***P***^**-**^**(MPa)**	***I***_**SPPA.3**_**(W/cm**^**2**^**)**
Clinical lithotripter	0.5	5	0.001 for 60 min	37–115	-10	20,000
Diagnostic US	2	1	0.01–1 continuous	4	3	190
Clinical simulation	2	100	3.3% in 1 s bursts	20	-10	2,400
Injury threshold	2	100	3.3% for 10 min	96	-16	16,620

Above the threshold intensity at the 3.3% duty cycle, the injury was found to range from individual cell necrosis to frank emulsification of the tissue with focally extensive hemorrhage. Only two samples below the threshold displayed hemorrhaging, and these instances of hemorrhage were at the surface of the kidney. It is possible that these injuries were caused by poor transducer coupling, or tissue-handling trauma, which would not occur in the clinical setting as treatment would be performed transcutaneously. It is important to note that both these cases occurred above 6,030 W/cm^2^, 2.5 times the intensity used in the clinical simulation to move kidney stones. Although both gross surface changes and focal hyperechogenicity during the exposure tracked with the histological injury patterns were observed, the proportions were slightly higher than observed histologically, particularly close to the calculated histological threshold. Again, it is possible that these events could have been at the surface or in the coupling to the tissue that would not be present in clinical use, but suggest that both gross surface changes and focal hyperechogenicity may occur before histological injury is observed.

When treatment was performed continuously for 10 min, the threshold for injury was found to be at 470 W/cm^2^. This low threshold could be due to the nature of the plateau model used to calculate the change point that identifies a single primary change point. It is possible that another change point occurs between 4,090 and 6,030 W/cm^2^, as there is a large jump in the proportion of samples, showing injury between these intensities (double). On looking at the quality of tissue injury, the second change point appears to be the true threshold for injury, whereas the original arises from randomly low injury in this control or lowest exposure data set. However, not enough information is available to evaluate these differences statistically. The observation of a low threshold for injury during continuous operation is a clear indication that ultrasonic propulsion has the potential to be injurious and that the system must be used in brief bursts such as performed in our clinical simulation [5]. Further, it is highly unlikely that this technology could be inadvertently misused in this way, given that continuous energy output would interfere with imaging and would be observed early in the treatment. In addition, many instruments designed to create pulses, often by charging a capacitor, would not be capable of producing a continuous sustained output.

This study suggests that duty cycles greater than 20% at a spatial peak intensity of 9,320 W/cm^2^ would be needed before the probability of injury rises above 0.3. Although this intensity approximates the maximum that could be achieved by an unmodified clinical prototype at a 4-cm focus without attenuation from tissue, this intensity is approximately four times greater than the *in situ* intensity that was used to effectively move stones in pigs. In all cases, 10 min at a steady duty cycle and focal location is significantly more US bursts than would be used clinically as the operator would need time between bursts to reacquire the stone and reposition the transducer. In the clinical simulation, the average procedure time was approximately 14 min, which corresponds to an average delay time of 41 s between bursts [5,13].

The types of injury observed at high outputs are consistent with those seen in SWL and other focused ultrasound therapies [13]. Overall, the results support earlier reports that injury is not seen at the levels used to reposition kidney stones [5,13]. There is room to adjust the intensity, duty cycle, number of bursts, and exposure duration without observing injury. As the peak pressure of the clinical prototype is one half that commonly used in SWL and the total energy delivered is less than one fourth [5], these results are also consistent with those of the previous reports with SWL outputs, where reductions of 10%–20% in peak pressure and 20%–50% in energy from standard lithotripsy eliminate measureable anatomic injury [12].

A limitation of this study is the procedure used to access the kidneys for treatment by direct contact with the US probe. For future clinical application, treatments will be performed transcutaneously, as was the method used in our clinical simulation [5]. In the current study, surgical access was chosen to ensure localization of the treated site, fine control of the exposure levels, and optimal utilization of the kidney tissue (up to seven lesions could be created in one kidney). When performed on an intact subject aberration of the beam, and more importantly, breathing motion, is likely to spread the acoustic energy over a larger volume of tissue and thus reduce the likelihood of injury. Future preclinical transcutaneous studies will need to address the potential of collateral injury to adjacent tissues, but given the dose levels proposed, this is highly unlikely.

Though the system in this study is different than the prototype, there are enough similarities between the systems to see that the identified injury threshold is far above the output levels capable of the prototype system. The acoustic data presented here are for intensity only; other parameters are reported elsewhere [5]. A limitation of this presentation include the fact that intensity does not account for non-linear acoustic effects, which can affect heating, such that different pulse shapes with a similar intensity can potentially cause different forms of thermal injury. Still, for the purpose of evaluating conditions relevant to propulsion of kidney stones, discussions in terms of intensity are appropriate.

## Conclusion

This preclinical exploratory study helps establish the margins of safety associated with the use of focused ultrasound for renal calculi displacement. Consequential injury only occurred with treatment conditions that far exceeded the dose needed to displace stones from the kidney. These settings are not even possible with the current clinical prototype. Thus, ultrasound to reposition kidney stones has the potential to be safe and effective.

## Abbreviations

H&E: Hematoxylin and eosin; HIFU: High-intensity focused ultrasound; NADH-d: Nicotinamide dinucleotide diaphorase; PAS: Periodic acid-Schiff; SWL: Shockwave lithotripsy; US: Ultrasound.

## Competing interests

The authors declare that they have no competing interests.

## Authors’ contributions

YNW participated in the study design; performed the *in vivo* study, histological processing and analysis, and data analysis; and drafted the manuscript. JCS participated in the study design and performed the *in vivo* study, transducer characterization, transducer calculations, and data analysis. BWC built the eight-element array system and developed the equipment for treatment application. FLS participated in the study design, performed surgeries, and established techniques for perfusion of the kidneys. MP performed all ultrasound-guided targeting and analyzed the ultrasound images in real time for changes (e.g., hyperechoic regions). DL participated in the study design, established the injury scoring rubric, and performed histological analysis and interpretation of injury. APE participated in the study design, guided tissue processing techniques, and performed histological analysis and interpretation of injury. JAM participated in the study design, performed histological analysis and interpretation of injury, and helped draft the manuscript. ZL developed and performed the statistical analysis. BD participated in the development of treatment protocol and correlation to the clinical simulation. MRB conceived of the study and participated in its design and coordination, performed the *in vivo* studies, analyzed the data, and helped draft the manuscript. All authors read and approved the final manuscript.
